# Interprofessional collaboration in Austrian primary care: an analysis of requirements and challenges: Melanie Roth

**DOI:** 10.1093/eurpub/ckac130.146

**Published:** 2022-10-25

**Authors:** A Atalaia, CJ Schnegg, B Grabner, M Roth

**Affiliations:** Department of Health Sciences, Salzburg University of Applied Sciences, Puch/Salzburg, Austria

## Abstract

**Background:**

Primary health care (PHC) is built on interprofessional collaboration (IPC) between health and social professions. According to international frameworks, interprofessional communication, client-centred care, collaborative leadership, role clarification, team functioning and interprofessional conflict resolution are essential for IPC. As of April 2022, 36 PHC units were registered in Austria. This study aims to explore the perspectives of health care professionals on IPC in PHC in Austria.

**Methods:**

Between October 2021 and March 2022, 15 guided group interviews were conducted with a total of 57 representatives of the professional groups biomedical analytics, dietetics, medical training therapy, medicine, midwifery, nursing, occupational therapy, office assistance, orthoptics, pharmacy, physiotherapy, psychotherapy, radiology technology, social work and speech therapy. The data was analysed using qualitative content analysis according to Mayring.

**Results:**

Interdisciplinary counselling and prevention services, monitoring, acute interventions and treatment of chronic diseases are seen as fields of action for increased IPC in PHC. Interprofessional relationships are established during joint home visits and weekly multiprofessional meetings, when communication is collegial. Challenges mentioned for the IPC in PHC were i.e. role ambiguity, lack of time for networking or unclear legal regulations. Taking over responsibility as well as the ability to delegate and to deal with conflict, a sense of justice and willingness to accept criticism are core competences required for IPC in PHC.

**Conclusions:**

The interviewees aim to get more involved in PHC, but not all feel optimally prepared for the necessary collaboration in this setting. In their view, specific training content, focusing on the unique structure of PHC, the roles of all the involved professional groups and conflict management, is necessary to successfully shape IPC in the interest of the clients.

**Key messages:**

- Austrian health care professionals desire specific training content for interprofessional collaboration in primary health care.
- Austrian health care professionals see opportunities but also challenges for their professional group in primary health care.

